# Predictors of restenosis after drug-coated balloon angioplasty for femoropopliteal chronic total occlusion lesions

**DOI:** 10.1186/s42155-025-00612-4

**Published:** 2025-10-28

**Authors:** Yuki Shima, Mihoko Sato, Gakuto Bando, Narumi Irie, Kazunori Mushiake, Naoya Inoue, Hiroyuki Tanaka, Kazushige Kadota

**Affiliations:** 1 Department of Cardiovascular Medicine, Kurashiki Central Hospital, 1-1-1 Miwa, Kurashiki, 710-8602 Japan; 2 Department of Clinical Laboratory, Kurashiki Central Hospital, 1-1-1 Miwa, Kurashiki, 710-8602 Japan

**Keywords:** Peripheral artery disease, Endovascular therapy, Drug-coated balloon, Intravascular ultrasound

## Abstract

**Background:**

Drug-coated balloons (DCBs) are widely used in endovascular therapy. While dissection angle and minimum lumen area (MLA) assessed by intravascular ultrasound (IVUS) are known predictors of restenosis, their specific role after DCB angioplasty remains to be fully elucidated. We aimed to identify predictors of restenosis following DCB angioplasty using IVUS findings.

**Methods:**

We retrospectively enrolled 36 peripheral artery disease patients undergoing DCB angioplasty (Jan 2021–Dec 2023). We evaluated IVUS images post-guidewire and post-DCB at 3-cm intervals, classifying cross-sections by MLA/external elastic membrane area (EEMA) ratio: > 50%, 40%–50%, and < 40%. Primary patency at 1 year post-DCB was the primary outcome. Restenosis was objectively determined by a peak systolic velocity ratio of 2.4 on duplex ultrasound, and assessing each cross-sectional images.

**Results:**

A total of 262 cross-sectional images were acquired and subsequently classified into three distinct groups based on their MLA/EEMA ratio: > 50% (*n* = 125), 40%–50% (*n* = 85), and < 40% (*n* = 52). All guidewires passed through the intraplaque route. Primary patency was significantly higher in the MLA/EEMA > 50% group (94.0% vs. 84.2% vs. 73.3%, log-rank *p* = 0.005). Specifically, for dissection angles > 60°, patency was markedly better in the MLA/EEMA > 50% group (93.3% vs. 75.0% vs. 55.6%, log-rank *p* = 0.03). Dissection angles < 60° showed no significant patency differences (93.9% vs. 88.0% vs. 84.2%, log-rank *p* = 0.14).

**Conclusions:**

The MLA/EEMA ratio and the degree of dissection angle may be predictors of primary patency following DCB angioplasty. These findings suggest that optimized vessel preparation strategies can effectively mitigate the adverse clinical impact of dissection.

## Introduction

Contemporary clinical guidelines advocate for the utilization of drug-coated balloon (DCB) angioplasty in the management of femoropopliteal lesions [1, 2]. Commendable patency rates have been documented following DCB angioplasty, extending even to instances of chronic total occlusion (CTO), which represents a particularly intricate lesion morphology [3, 4]. Within the specific context of CTO lesions, intraluminal DCB angioplasty has demonstrated superiority compared to subintimal approaches [5]. Consequently, the judicious selection of an appropriate guidewire route is paramount for the achievement of sustained primary patency and the prevention of severe iatrogenic dissection in CTO lesions [6].

Intravascular ultrasound (IVUS) serves as a potent diagnostic modality, furnishing critical information such as vessel diameter, guidewire trajectory, dissection morphology, calcification burden, and postprocedural luminal dimensions, thereby constituting an indispensable asset for enhancing endovascular therapy (EVT) outcomes. Prior research has indicated that a greater minimum lumen area (MLA), as quantitatively assessed by IVUS following DCB angioplasty, is associated with a reduced incidence of restenosis [7]. Nevertheless, the magnitude of the MLA is intrinsically modulated by vessel diameter and the extent of calcification, both established predictors of restenosis after DCB angioplasty [7]. Moreover, the dissection angle has been demonstrated to correlate with primary patency rates subsequent to DCB angioplasty [8, 9]. The purpose of this study was to elucidate the comprehensive predictors of restenosis following DCB angioplasty, drawing upon detailed IVUS findings.

## Methods

### Study population and design

We conducted a single-center, retrospective cohort study of 36 patients with peripheral artery disease defined as Rutherford class 2–5. All patients underwent initial EVT with DCB angioplasty for femoropopliteal CTOs from January 2021 to December 2023, using Ranger (Boston Scientific Corporation, Marlborough, MA, USA) or IN.PACT Admiral (Medtronic Vascular, Santa Clara, CA, USA) DCBs. IVUS images, acquired post-guidewire and post-DCB, were analyzed at 3-cm intervals. Cross-sectional images were stratified into three groups based on the MLA/external elastic membrane area (EEMA) ratio: > 50%, 40%–50%, and < 40%. Patients had symptomatic atherosclerosis from the superficial femoral artery to the proximal (P1) popliteal artery. Exclusion criteria were absence of atherosclerosis, Rutherford class 0 or 6, mid (P2) or distal (P3) popliteal lesions, life expectancy < 1 year, advanced malignancy, and acute limb ischemia. All patients provided informed consent. The study received institutional ethics committee approval.

### Procedural protocol

In this study, three experienced operators, who had performed more than 500 cardiovascular interventions each, performed all procedures. EVT was executed via ipsilateral or contralateral approach originating from the common femoral artery, utilizing a 5-F or 6-F guiding sheath. Unfractionated heparin served as the anticoagulant agent. Antegrade access was achieved employing 0.014-, 0.018-, or 0.035-inch guidewires with adjunct support catheters; a retrograde approach was implemented as clinical necessity dictated. Following guidewire passage, the trajectory of the guidewire was meticulously monitored via IVUS; in instances where a subintimal route was identified, intraplaque wiring was subsequently performed. The categorization of guidewire routes (i.e., intraplaque, subintimal, or intramedial) was rigorously established based on IVUS findings [10]. Predilation was universally conducted in all patients, guided by either angiographic or IVUS observations. The entirety of the lesion was initially dilated with a smaller 3.0- or 4.0-mm balloon, succeeded by predilation with an optimally sized balloon. Contingent upon IVUS findings, supplementary balloon dilation was performed using either a scoring balloon or a high-pressure balloon. The duration of dilation was primarily at the operator’s discretion but maintained a minimum of one minute for all patients. DCB angioplasty was undertaken only after confirming, to the maximum extent feasible that the residual stenosis remained below 50% and the degree of dissection was inferior to grade D [11, 12]. The selection of devices for EVT and the subsequent postprocedural management, including antithrombotic regimens such as antiplatelets or anticoagulants, were determined by the discretion of the treating physician. The recommended antiplatelet therapy comprised long-term aspirin (100 mg/day) in conjunction with clopidogrel (75 mg/day) and/or cilostazol (100 or 200 mg/day) [13]. Dual antiplatelet therapy was maintained for a minimum duration of 1 month post-EVT.

### Study endpoints and definitions

The primary outcome measure established for this study was 1-year primary patency, defined as the absence of restenosis. Restenosis was objectively determined by a peak systolic velocity ratio of 2.4 on duplex ultrasound. Evaluation of target vessel patency was conducted at 6-month intervals for patients presenting with claudication. For patients afflicted with chronic limb-threatening ischemia, follow-up assessments were performed more frequently, ranging from monthly to every several months, primarily owing to the imperative for consistent wound assessment over a 1-year period. Patients with claudication specifically underwent follow-up examinations s at 6 and 12 months subsequent to their EVT. Examinations were performed duplex ultrasound and ankle brachial index, and assessing each cross-sectional images.

**Fig. 1:**
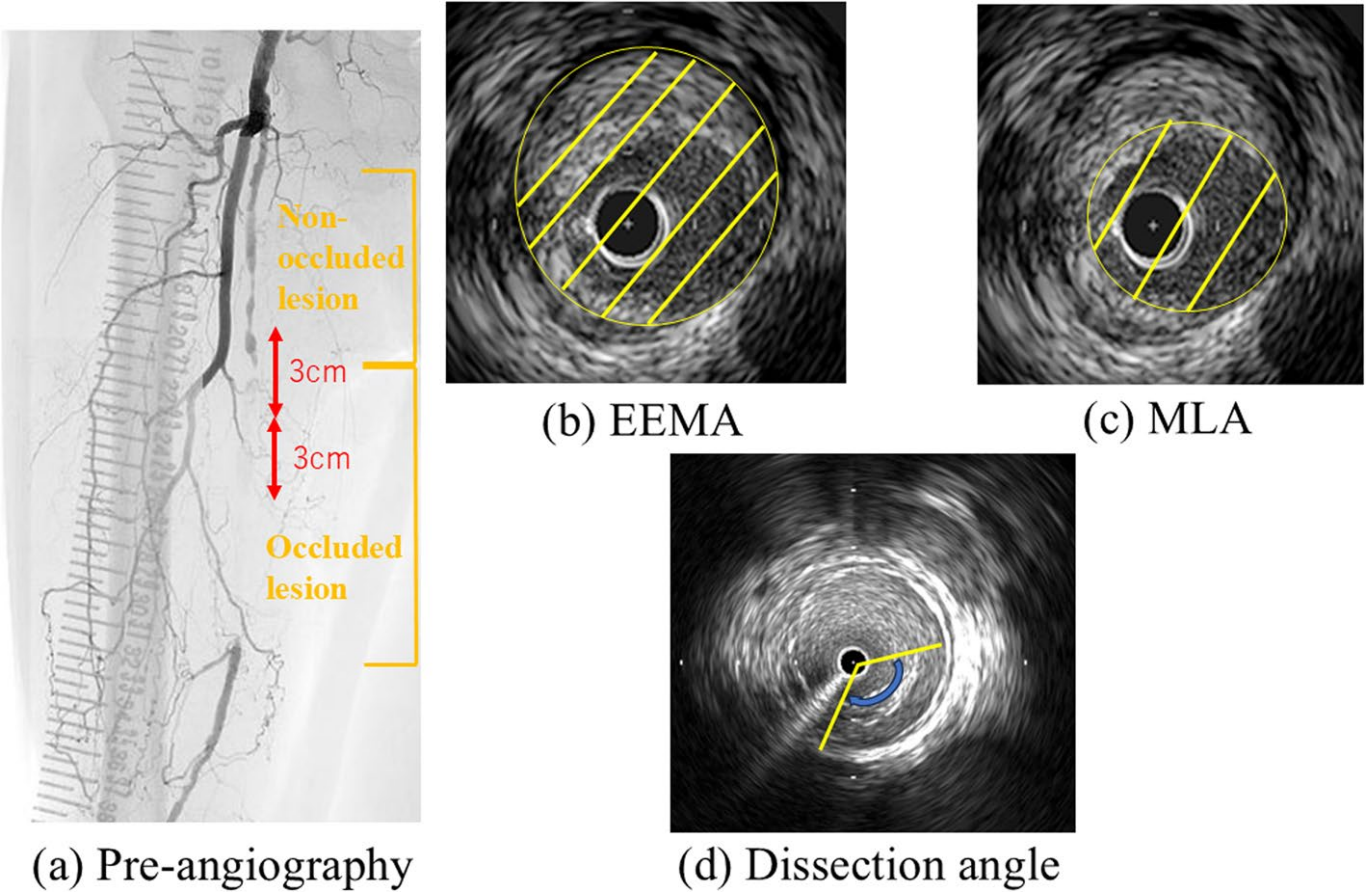
**a** Pre-angiography. IVUS images were analyzed at 3-cm intervals. **b** IVUS image and measurement of EEMA. **c** IVUS image and measurement of MLA. **d** IVUS image and measurement of dissection angle. IVUS, intravascular ultrasound; EEMA, external elastic membrane area; MLA, minimum lumen area

IVUS examinations, encompassing both baseline and postprocedural assessments, were performed utilizing the AnteOwl system (Terumo, Tokyo, Japan), with image acquisition achieved via automated pullback through the target lesion. EEMA was precisely quantified at the corresponding site of MLA. Figure 1 shows the details of definitions in this study.

Two independent, experienced cardiologists (Y.S. and N.T.), who remained blinded to all clinical and procedural data, meticulously classified all relevant IVUS parameters based on their individual evaluations and subsequent mutual consensus. In instances of divergence, a definitive opinion was rendered by a third consulting cardiologist (K.M.). All parameter analyses strictly conformed to the guidelines outlined in the current clinical expert consensus document concerning EVT imaging [14]. The dissection angle was measured using an electronic protractor centered on the lumen. These findings were measured by manually.

### Statistical analysis

Categorical variables were subjected to comparison utilizing the chi-square test. Continuous variables, quantitatively expressed as mean ± SD, underwent comparative analysis using Student’s t-test or the Wilcoxon rank-sum test, contingent upon their distributional properties. The Kolmogorov–Smirnov test was rigorously applied to ascertain the normality of the distribution for all quantitative variables. Cumulative incidence was estimated employing the Kaplan–Meier method, with differences rigorously assessed via the log-rank test. A *p*-value of < 0.05 was established as the threshold for statistical significance. All statistical computations were executed using JMP version 18.0 software (SAS Institute, Cary, NC, USA).

## Results

### Baseline characteristics

Table 1 delineates the baseline characteristics of the study cohort. A total of 262 cross-sectional images were acquired and subsequently classified into three distinct groups based on their MLA/EEMA ratio: > 50% (*n* = 125), 40%–50% (*n* = 85), and < 40% (*n* = 52). The corresponding baseline patient and lesion characteristics, stratified by these cross-sectional image classifications, are comprehensively summarized in Table 2. Comparative analysis across the three MLA/EEMA ratio groups revealed no statistically significant differences in the prevalence of hemodialysis (28.8% vs. 17.7% vs. 25.0%, *p* = 0.39). However, there were significant differences in the prevalence of chronic limb-threatening ischemia (47.2% vs. 30.6% vs. 36.5%, *p* = 0.047) or diabetes mellitus (48.0% vs. 43.5% vs. 65.4%, *p* = 0.04). Moreover, a significantly elevated rate of occluded lesions was observed within the MLA/EEMA < 40% group (45.6% vs. 63.5% vs. 75.0%, *p* < 0.001).

**Table 1:** Baseline demographic and clinical characteristics of patients with femoropopliteal chronic occlusion lesions

Patients, n	36
Age, yrs	79.8 ± 7.5
Males, n (%)	25 (69.4)
BMI	21.8 ± 3.8
Hypertension, n (%)	29 (80.5)
Diabetes mellitus, n (%)	17 (47.2)
Hyperlipidemia, n (%)	21 (58.3)
Hemodialysis, n (%)	8 (22.2)
Smoking history, n (%)	19 (52.7)
CLTI, n (%)	13 (36.1)
BTK runoff vessels (n)	2.09 ± 0.75
Lesion length (mm)	210.2 ± 73.9
Occlusion length (mm)	109.6 ± 78.5

Values are expressed as mean ± standard deviation (SD) or percentage (%), as indicated. *BMI* body mass index, *CLTI* chronic limb-threatening ischemia, *BTK* below-the-knee

**Table 2:** Patient and lesion characteristics by cross-sectional image analysis of femoropopliteal chronic occlusion lesions

	MLA/EEMA ratio > 50%	MLA/EEMA ratio of 40% to 50%	MLA/EEMA < 40%	p
Sections, n	125	85	52	
Age	74.1 ± 6.5	77 ± 6.5	78.2 ± 7.0	0.002
Males, n (%)	81 (64.8)	60 (70.1)	33 (63.4)	0.32
Hypertension, n (%)	103 (82.4)	72 (84.7)	43 (82.7)	0.9
Diabetes mellitus, n (%)	60 (48.0)	37 (43.5)	34 (65.4)	0.04
Hyperlipidemia, n (%)	71 (56.8)	60 (70.6)	37 (71.2)	0.06
Smoking history, n (%)	83 (66.4)	50 (58.8)	25 (48.1)	0.07
Hemodialysis, n (%)	36 (28.8)	15 (17.7)	13 (25.0)	0.18
Occluded lesion, n (%)	57 (45.6)	54 (63.5)	39 (75.0)	< 0.001
Occluded length, mm	22.1 ± 9.7	24.8 ± 8.1	24.2 ± 8.9	0.24
Lesion length, mm	26.8 ± 6.5	26.6 ± 6.7	26.4 ± 7.1	0.93
CLTI, (%)	59 (47.2)	26 (30.6)	19 (36.5)	0.04
MLA, mm^2^	15.5 ± 3.9	13.3 ± 2.9	11.1 ± 2.6	< 0.01
EEMA, mm2	26.9 ± 6.8	29.9 ± 6.7	36.4 ± 6.8	0.002
EEM < 5 mm, n (%)	15 (12.0)	5 (5.9)	3 (5.8)	0.21
EEM 5–6 mm, n (%)	53 (42.4)	29 (34.1)	14 (26.9)	0.13
EEM > 6 mm, n (%)	57 (45.6)	51 (60.0)	34 (65.4)	0.02
Dissection angle > 60°, n (%)	31 (24.8)	25 (29.4)	17 (32.7)	0.53
Severe calcification > 270°, n (%)	15 (12.0)	13(15.3)	9 (17.3)	0.6
INPACT DCB, n (%)	65 (52.0)	38 (44.7)	26 (50.0)	0.58
Ranger DCB, n (%)	60 (48.0)	47 (55.3)	26 (50.0)	0.58
Bailout stenting, n (%)	0 (0)	0 (0)	0 (0)	

Data are shown as mean ± standard deviation or percentage (%), unless noted. *CLTI* chronic limb-threatening ischemia, *MLA* minimum lumen area, *EEMA* external elastic membrane area, *EEM* external elastic membrane, *DCB* drug-coated balloon

### Outcome measures

All guidewires passed through the intraplaque route. Across the three comparative groups, the mean EEMA was significantly larger within the MLA/EEMA < 40% cohort (26.9 mm^2^ vs. 29.9 mm^2^ vs. 36.4 mm^2^, *p* = 0.02). Commensurately, the proportion of vessels exhibiting a large diameter (EEM > 6 mm) was also significantly elevated in the MLA/EEMA < 40% group (45.6% vs. 60.0% vs. 65.4%, *p* = 0.02). Conversely, no statistically significant disparities were observed concerning the degree of dissection (29.0° vs. 37.3° vs. 43.3°, *p* = 0.25) or the prevalence of severe calcification (12.0% vs. 15.3% vs. 17.3%, *p* = 0.61).

**Fig. 2:**
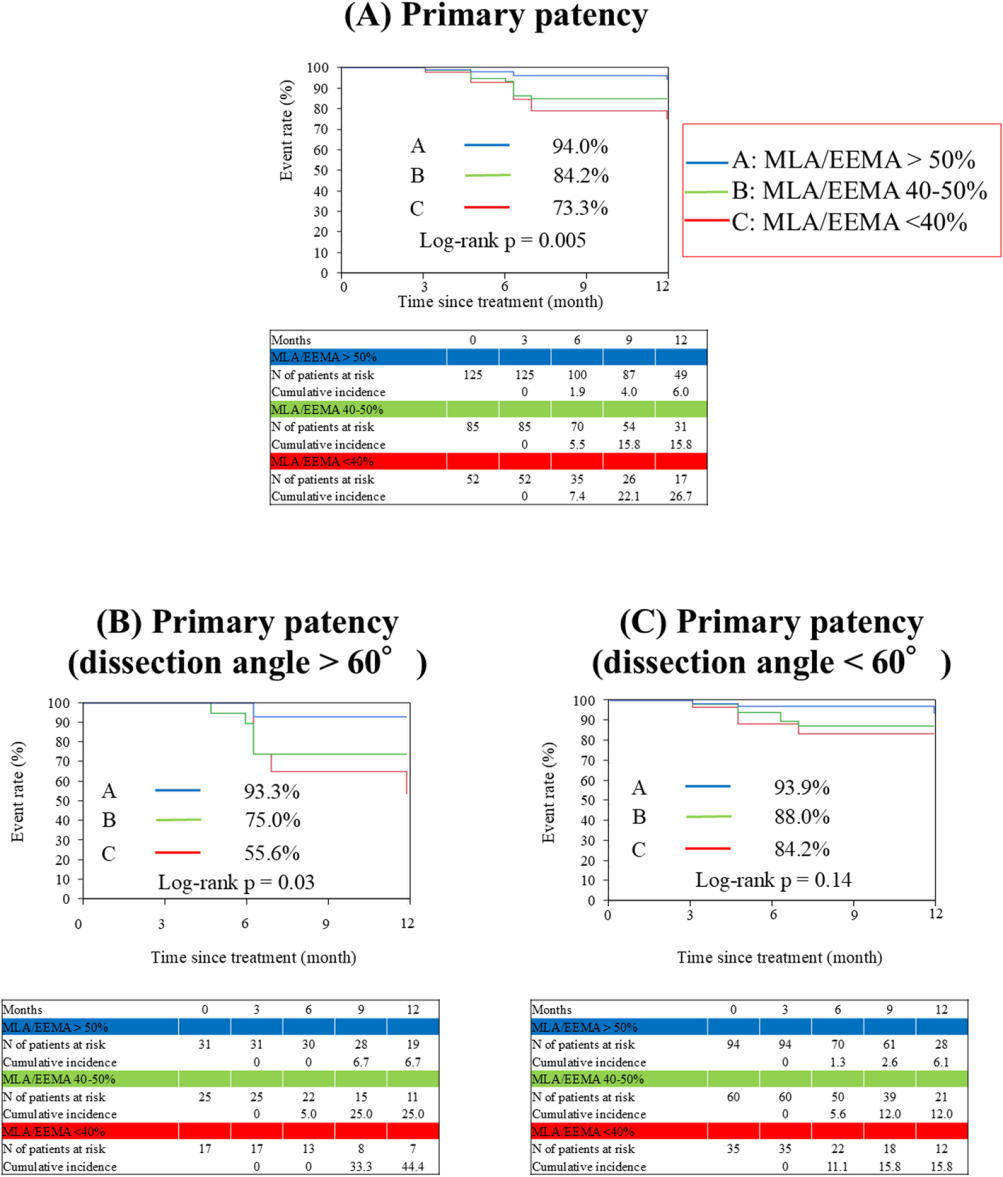
Kaplan–Meier curves for 1-year primary patency following drug-coated balloon angioplasty. **A** Overall primary patency. **B** Primary patency in cross-sections with a dissection angle > 60 degrees. **C** Primary patency in cross-sections with a dissection angle < 60 degrees

As presented in Fig. 2, Kaplan–Meier estimations illustrate the cumulative primary patency rates. Primary patency was demonstrably and statistically significantly higher in the MLA/EEMA > 50% cohort compared to the 40%–50% and < 40% groups (94.0% vs. 84.2% vs. 73.3%, log-rank *p* = 0.005). Further stratification by dissection angle revealed that in cross-sectional images with a dissection angle exceeding 60 degrees, the patency rate remained significantly elevated in the MLA/EEMA > 50% group (93.3% vs. 75.0% vs. 55.6%, log-rank *p* = 0.03). Conversely, for cross-sectional images characterized by a dissection angle of less than 60 degrees, no statistically significant disparity in primary patency was discernible across the three groups (93.9% vs. 88.0% vs. 84.2%, log-rank *p* = 0.14).

## Discussion

The main findings derived from this investigation are as follows: (1) the attainment of a MLA/EEMA ratio > 50% consistently portends favorable primary patency, even within the challenging context of CTO lesions; (2) optimal patency can be anticipated even in the presence of dissection, provided the MLA/EEMA ratio exceeds 50%. Conversely, a limited dissection angle may attenuate the influence of residual lumen area on long-term patency.

Recent investigations by Ko et al. have elucidated that IVUS-guided EVT significantly enhances the clinical outcomes of DCB angioplasty. The discernible advantages of IVUS guidance encompass superior detection of calcifications and arterial dissections, coupled with the capability for precise quantification of vessel dimensions and the identification of optimal landing zones [15]. Furthermore, Kurata et al. have reported a significant association between DCBs sized according to IVUS-EEM dimensions, as opposed to angiographic lumen or IVUS-lumen dimensions, and a diminished risk of restenosis following EVT for femoropopliteal lesions [16]. Specifically, a larger MLA has been posited to correlate with a reduced incidence of restenosis at 1 year post-DCB angioplasty [7]. Furthermore, Kozuki et.al reported that IVUS-evaluated post-procedural dissection angle was associated with restenosis at 1-year follow-up after femoropopliteal EVT with DCB [8]. In congruence with these prior observations, the present study demonstrates robust 1-year primary patency within the MLA/EEMA > 50% cohort, juxtaposed with suboptimal patency in the MLA/EEMA < 40% group. Given that residual stenosis constitutes an independent predictor of restenosis within 1 year following DCB angioplasty [2], the judicious selection of an appropriate balloon based on comprehensive IVUS measurements is demonstrably paramount.

Although previous reports have posited an association between primary patency and MLA, dissection angle, and calcification [7, 8, 17], a comprehensive evaluation of these factors in combination has been absent. To the best of our knowledge, the present investigation represents the first study to synergistically assess IVUS findings following DCB angioplasty. Our findings indicate that the impact of dissection on patency is attenuated when an adequate lumen area is achieved, yet becomes pronounced when optimal lumen gain is not attained. Importantly, favorable patency outcomes can still be anticipated even with a suboptimal lumen area if the associated dissection angle is minimal. It is necessary to minimize the angle of dissection, and the appropriate selection of wires and balloon expansion strategies is essential to prevent severe dissection (classified as Type D or higher according to the National Heart, Lung, and Blood Institute classification). Central wiring, employed intraluminally and confirmed by IVUS in all lesions in this study, can effectively preclude the formation of severe dissections. Furthermore, during balloon dilation, careful consideration should be given to extended inflation times and the utilization of scoring balloons, as these interventions have been suggested to reduce the risk of severe dissection [18, 19]. Our DCB angioplasty protocol used IVUS-measured distal reference vessel diameters for balloon sizing, aiming to prevent vessel over-injury. This strategy, however, may lead to under-dilation of larger proximal segments in long lesions, potentially explaining the greater proportion of larger EEMs in the MLA/EEMA < 40% group. Optimal patency may require adequately dilating these proximal vessel portions. While severe calcification is a known restenosis predictor, we did not observe this in our cohort. This could be due to successful lumen gain in many cases, but warrants further investigation in longer-term studies.

Recently, Swedepad 2 trial reported that patients with Rutherford stage 1–3 undergoing EVT with paclitaxel-coated devices did not improve disease-specific quality of life at 1 year and all-cause mortality was higher over 5 years compared with uncoated devices [20]. However, VAYAGER PAD trial reported that patients with PAD who underwent EVT with paclitaxel-coated devices were not associated with mortality or major adverse limb events but were associated with persistent reduction in unplanned index limb revascularization [21]. In addition, Nakamura et al. reported that meta-analysis of data from 2,581 Japanese cases obtained in randomized controlled trials and single-arm studies found no harmful effect on 5-year mortality with the use of paclitaxel-coated devices for symptomatic femoropopliteal artery disease [22]. The clinical outcomes of paclitaxel-coated device remain unclear, further evaluation is needed.

### Limitations

Our study presents four primary limitations. First, its single-center, retrospective, observational nature and small patient sample raise concerns about selection bias affecting the conclusions. Second, operator discretion in DCB angioplasty, without a predefined protocol, may have introduced selection bias. Third, experienced cardiologists, not an external core laboratory, interpreted angiographic and IVUS findings. We mitigated measurement bias by adhering to the current consensus document [14]. Lastly, we did not utilize atherectomy devices.

## Conclusions

The MLA/EEMA ratio and the degree of dissection angle may be independent predictors of primary patency. Furthermore, these findings suggest that meticulous vessel preparation strategies are instrumental in minimizing the detrimental consequences of dissection.

## Data Availability

The datasets used and/or analyzed during the current study are available from the corresponding author upon reasonable request.

## Abbreviations

DCB: Drug-coated balloon
CTO: Chronic total occlusion (CTO)
IVUS: Intravascular ultrasound
EVT: Endovascular therapy
MLA: Minimum lumen area
EEMA: External elastic membrane area
